# A novel mutation in the MIP gene is associated with autosomal dominant congenital nuclear cataract in a Chinese family

**Published:** 2011-05-13

**Authors:** Guoxing Yang, Guisen Zhang, Qiang Wu, Jialiang Zhao

**Affiliations:** 1 Department of Opthalmology, Peking Union Medical College Hospital, Chinese Academy of Medical Sciences & Peking Union Medical College, Beijing, China; 2 Department of Opthalmology, Red Cross Society of Inner Mongolia Chao Ju Ophthalmic Hospital, Inner Mongolia Autonomous Region, China

## Abstract

**Purpose:**

Congenital cataracts are a clinically and genetically heterogeneous lens disorder. The purpose of this study was to identify the genetic mutation and the molecular phenotype responsible for the presence of autosomal dominant congenital nuclear cataract disease in a Chinese family.

**Methods:**

Patients were given physical examinations and their blood samples were collected for DNA extraction. Genotyping was performed by microsatellite markers and logarithm-of-odds (LOD) scores were calculated using the LINKAGE programs. Mutation detection was performed by direct sequencing.

**Results:**

Linkage to the major intrinsic protein (*MIP*) locus was identified. Sequencing *MIP* revealed an A→G transition at nucleotide position c.530, which caused a conservative substitution of Tyr to Cys at codon 177 (P.Y177C). The Y177C mutation is located in the fifth transmembrane sequence. This mutation was identified in all affected individuals but is not found in any of the 100 control chromosomes.

**Conclusions:**

Our results identify that the c.530 (A→G) mutation in *MIP* is responsible for the Chinese pedigree. Our results further identify that the mutation in *MIP* is responsible for congenital cataract. The mutation found in our study broadens the spectrum of *MIP* mutations.

## Introduction

Congenital cataracts are common and cause approximately one-third of infant blindness; they occur in approximately 1–6 out of every 10,000 live births. One quarter of congenital cataract disease is hereditary [1-4].

Congenital cataracts are a clinically and genetically heterogeneous lens disorder. Cataracts that are phenotypically identical can result from mutations at different genetic loci and can have different inheritance patterns. Conversely, cataracts with dissimilar phenotypes may result from mutations in a single gene or gene family. It is believed that the type of genetic mutation is related to the morphology of the cataract [5]. To date, about 40 genetic loci have been linked to congenital cataracts and 26 genes have been cloned and sequenced, including crystallins, connexins, heat shock transcription factor-4, aquaporin-0, and beaded filament structural protein-2 [5].

Recently, we found a four-generation pedigree from northeast China with congenital nuclear cataract. Mutation screening in the major intrinsic protein gene (*MIP*) identified an A→G transition at nucleotide position c.530. This nucleotide change resulted in the substitution of highly-conserved Tyr by Cys at codon 177 (P.Y177C).

## Methods

### Patients and clinical data

The five-generation family enrolled in this study was found in a Northeastern province of China. Clinical examination, peripheral blood collection and DNA extraction were performed in the Department of Ophthalmology, Peking Union Medical College Hospital, Beijing, China. The study was performed in accordance with the Declaration of Helsinki and approved by the Institutional Review Board and Ethics Committee of Peking city; informed consent was obtained from all participants.

Recruited family members included 7 confirmed patients with congenital nuclear cataract. Blood was collected from 6 patients and 5 unaffected family members. Clinical data for these 11 subjects was ascertained by detailed ocular examinations.

### Genotyping and linkage analysis

DNA was isolated from the blood samples of the 11 individuals and fluorescently-labeled microsatellite markers were used for linkage analysis of 26 candidate gene regions. Two-point linkage analysis was performed with MLINK from the LINKAGE program package.

### Mutation analysis

All coding exons of *MIP* were amplified by polymerase chain reaction (PCR) using a set of four pairs of primers [6], shown in Table 1. The PCR products were sequenced on an ABI3730 Automated Sequencer (PE Biosystems, Foster City, CA).

**Table 1:** Primers used for PCR amplification of *MIP*.

**Exon**	**primer (5′-3′)**	**Product length (bp)**	**Annealing temperature (°C)**
MIP-1F	GACTGTCCACCCAGACAAGG		
MIP-1R	TCAGGGAGTCAGGGCAATAG	492	58
MIP-2F	TGAAGGAGCACTGTTAGGAGATG		
MIP-2R	AGAGGGATAGGGCAGAGTTGATT	500	58
MIP-3F	CCAGACAGGGCATCAGT		
MIP-3R	TGGTACAGCAGCCAACAC	373	58
MIP-4F	AAGGTGTGGGATAAAGGAGT		
MIP-4R	TTCTTCATCTAGGGGCTGGC	429	58

Cited from reference [6].

## Results

### Clinical findings

**Figure 1:**
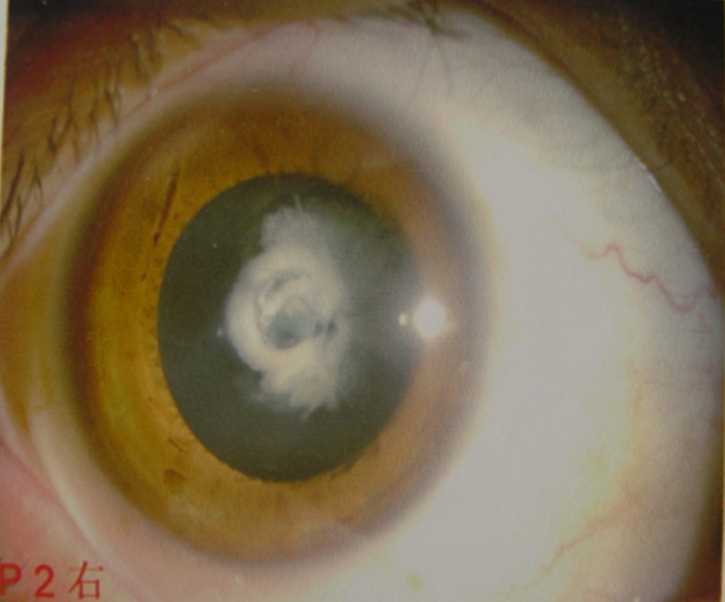
Slit lamp photograph showing nuclear cataract of patient IV:3 from Figure 2.

We identified a four-generation family with seven conformed individuals affected with congenital cataract (Figure 1). Blood was collected from six patients and five other family members. None of the participating patients have any other clinically-related ophthalmic syndromes.

### Linkage and haplotype analysis

**Figure 2:**
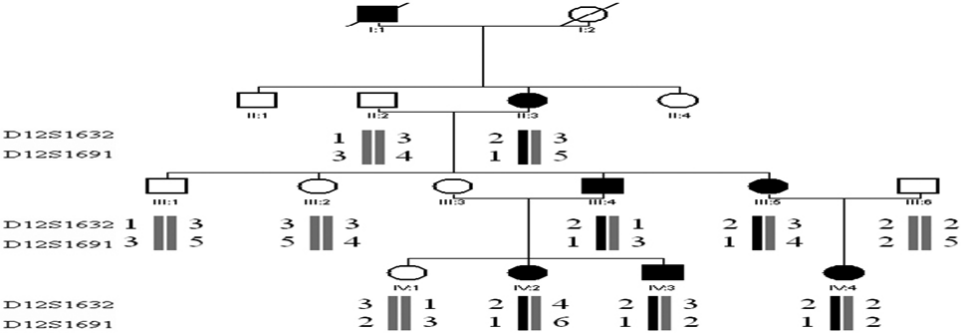
Pedigree and haplotype of the family. A four-generation pedigree with eleven available members is shown. Two markers (D12S1632 and D12S1691) close to *MIP* were used. The disease haplotype (represented by the black bar) cosegregated with all affected members but was not shared with any of the unaffected members.

We obtained positive logarithm-of-odds (LOD) scores with markers at 12q13 (Table 2). A maximum positive LOD score of 2.11 at θ=0.00 was obtained with marker D12S1632. The haplotype showed complete cosegregation in all six of the affected individuals analyzed (Figure 2).

**Table 2:** Result of linkage analysis.

**Marker**	**LOD scores at θ=**					
** **	**0.00**	**0.01**	**0.10**	**0.20**	**0.30**	**0.40**
D12S1632	2.11	2.07	1.74	1.33	0.88	0.41
D12S1691	2.11	2.07	1.74	1.33	0.88	0.41

### Mutation analysis

**Figure 3:**
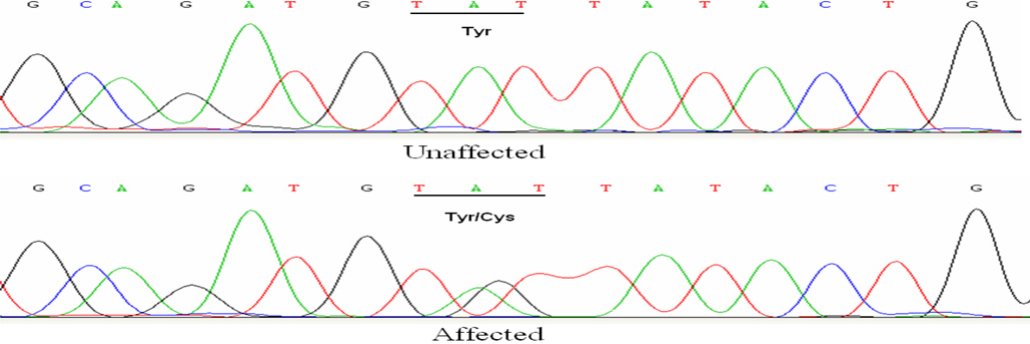
DNA sequences of *MIP* in unaffected and affected individuals. A heterozygous change A>G at codon 177 (UAU-UGU) resulted in the substitution of Tyr by Cys (P.Y177C) in the affected individuals.

By directly sequencing the coding region of *MIP*, a novel heterozygous A→G transition at nucleotide position 530 (c. 530 A→G) was detected in this family (Figure 3). This transition leads to the replacement of a highly-conserved Tyr to Cys at codon 177 (P.Y177C). Analysis of all other members of this family showed cosegregation of this change with the disease phenotype only in the affected individuals. This nucleotide substitution was not observed in any of the 100 control chromosomes from individuals with the same ethnic background.

## Discussion

Aquaporins are a large family. Since Agre demonstrated the water channel AQP1 in 1992, 13 isoforms have been found in mammals, including AQP0 to AQP12 [7]. Aquaporin expression is found throughout the body but is tissue-specific. Malfunctions of aquaporins are associated with diseases.

The *MIP* gene, also known as MIP26 or the AQP0 gene, encodes a 263-amino acid peptide. MIP is inserted in the plasma membrane with six transmembrane bilayer-spanning domains. Two NPA box domains form a water pore channel spanning the lipid bilayer. MIP exists as a tetramer in the plasma membrane [8,9].

Up to now, seven mutations have been reported, including c. 401A→G, c. 413C→G, c. 698G→A, c. 97C→T, c. 319G→A, IVS3–1 G→A, and 3223delG [6,8,10-13]. These mutations resulted in different phenotypes. The protein-functions study found that the mutated proteins affected water-permeability properties and trafficking [14,15].

The Y177C mutation is located in the fifth transmembrane. The substitution of Tyr by Cys would create an extra -SH that may result in incorrect disulfide bonds and influence the formation of a correct structure. The mutated protein may changes the water pore channel function, possibly through affecting water-permeability properties or trafficking. Further study of the protein function is needed to provide insights into the molecular mechanism of the mutation identified in this study.

In conclusion, we found a novel heterozygous Y177C mutation in *MIP* of an autosomal dominant congenital nuclear cataract family. These findings expand the mutation spectrum of *MIP*.
